# Risk factor control and outpatient attendance in young adults with diabetes

**DOI:** 10.1007/s00592-018-1261-5

**Published:** 2018-12-21

**Authors:** F. Nadeem, A. Urwin, M. Marshall, I. Doughty, H. Thabit, M. K. Rutter, L. Leelarathna

**Affiliations:** 1 School of Medicine, Faculty of Biology, Medicine and Health, University of Manchester, Manchester, UK; 2 Manchester Diabetes Centre, Manchester University NHS Foundation Trust, Manchester Academic Health Science Centre, Manchester, UK; 3 Royal Manchester Children’s Hospital, Manchester University NHS Foundation Trust, Manchester, UK; 4 Division of Diabetes, Endocrinology and Gastroenterology, Faculty of Biology, Medicine and Health, University of Manchester, Manchester, UK

**Keywords:** Type 1 diabetes, Type 2 diabetes, Young adults, Transition

## Introduction

Diabetes is one of the commonest chronic medical conditions amongst those aged 18–25 years. Type 1 diabetes (T1DM) predominates in this age group, but, recently, the prevalence of type 2 diabetes (T2DM) has also increased in young adults. Young people experience various social, psychological, and physiological changes, often leading to poor glycaemic control and non-engagement with health services [1].

Improving glycaemic control and controlling cardiovascular risk factors may reduce long-term complications [2]. This is particularly relevant for those who develop diabetes at a younger age due to its life-long nature and potential metabolic memory imprinting on vascular complications. We undertook a retrospective service evaluation in a large tertiary diabetes centre to assess glycaemic control, cardiovascular risk, and outpatient attendance of 18–25 years old.

## Methods

This was a retrospective, observational, single-centre, service evaluation in a tertiary referral centre in the North West of UK with approximately 1500 individuals with T1DM and 2000 individuals with T2DM. We included individuals aged 18–25 years who attended at least one medical appointment (*n* = 240).

As a service evaluation, no ethical approval was required. Data were collected from electronic records. HbA1c values were used from the most recent 12-month period at Manchester Diabetes Center. Data on weight, blood pressure (BP), and lipids were collected from the most recent visit. Non-attendance rates were calculated only after the exclusion of cancelled appointments. For each participant postcode, we obtained the English Index of Multiple Deprivation 2015 (IMD) from the Department for Communities and Local Government. The IMD is the official measure of relative deprivation for small areas in England, which ranks these areas from 1 (most deprived area) to 32,844 (least deprived area). We used IBM SPSS version 22 (IBM UK, Portsmouth) for statistical analysis.

## Results

Two hundred and forty individuals aged 18–25 years (at March 2016) attended Manchester Diabetes Centre between November 2007 and May 2016. Of these, follow-up data were available, HbA1c for 223 (93%), lipids for 219 (91%), BP for 223 (93%), and BMI for 214 (89%) individuals.

Baseline characteristics are shown in Table 1.

**Table 1:** Baseline characteristics

Characteristic	Data
*N*	240
Females	121 (50%)
Current age (year)	23 (21, 24)
Age at diagnosis (year)	12 (7, 16)
Duration of diabetes (year)	11 (5, 15)
Type 1 diabetes	209 (87%)
Type 2 diabetes	24 (10%)
Continuous subcutaneous insulin infusion (CSII)	78 (37%)^a^
IMD	8200 (3295, 13,149)

Data are *N* (%) or median (IQR) unless stated

*IMD* english index of multiple deprivation 2015

^a^Type 1 diabetes only

### Glycaemic control

For the whole cohort, the median number of HbA1c tests performed during the most recent 12-month period was 2 (1, 3). The median HbA1c level was 70 (57, 88) mmol/mol [8.6 (7.4, 10.2%)]. Females tended towards poorer glycaemic control; 72 (8.7%) vs. 68 (8.4%) mmol/mol, *p* = 0.052. Only 19% of the group had an HbA1c level ≤ 53 mmol/mol (7%). More than half of individuals (52%) had an HbA1c level > 69 mmol/mol (8.5%) and 26% had an HbA1c > 86 mmol/mol (10%) indicating poor or very poor glycaemic control.

Individuals with T1DM had significantly higher HbA1c compared to T2DM, 72 (8.7%) vs 49 (6.6%) mmol/mol, *p* < 0.001. Of those with T1DM, only 15% had an HbA1c level ≤ 53 mmol/mol (7%), 22% had HbA1c level ≤ 59 mmol/mol, and 56% with HbA1c ≥ 69 mmol/mol. Of individuals with T1DM (*n* = 209), 78 (37%) were treated with insulin pump therapy. There was no difference in median HbA1c between pump users and injection users, [75 (9.0%) vs. 70 (8.6%) mmol/mol, *p* = 0.15]. Longer duration of diabetes (*r* = 0.3, *p* < 0.001) was associated with higher HbA1c levels.

### Cardiovascular risk factor levels

Table 2 shows the median levels of HbA1c, blood lipids, BP, andbody mass index (BMI) separately for individuals with T1DM and T2DM separately. Less than one-third of the cohort with both types of diabetes had a total cholesterol (TC) < 4 mmols. LDL-cholesterol levels were broadly comparable in T1DM and T2DM, while more individuals with T1DM achieved HDL cholesterol targets. Triglyceride levels were numerically higher in those with T2DM. In contrast, BP was well controlled in the majority. Of the total cohort, 35% were overweight (BMI: 25–30 kg/m^2^) and 17% were obese (BMI > 30 kg/m^2^). Only 47% had an ideal BMI between 18 and 25 (in 2% BMI was < 18 kg/m^2^). For those with T1DM, 46% were overweight or obese (BMI ≥ 25 kg/m^2^). Taken as a whole, higher BMI was associated with higher LDL (*r* = 0.16, *p* = 0.026), lower high-density lipoprotein (HDL) (*r* = 0.26, *p* = < 0.001), higher triglycerides (*r* = 0.33, *p* < 0.001), higher systolic (*r* = 0.36, *p* < 0.001), and diastolic BP (*r* = 0.24, *p* < 0.001).

**Table 2:** Levels of cardiovascular risk factors and the proportion of individuals achieving treatment targets for type 1 and type 2 diabetes

	Treatment target	Type 1 diabetes			Type 2 diabetes		
		Median (IQR)	*N*	% Achieving target	Median (IQR)	*N*	% Achieving target
HbA1c	<=53 mmol/mol	72(60, 88)	192	15	49 (39, 68)	24	54
TC	< 4 mmol/L	4.7 (4.0, 5.1)	188	29	4.7 (4.0, 5.1)	24	25
LDL-C	< 2 mmol/L	2.4 (1.9, 2.9)	181	30	2.5 (1.7, 3.1)	22	32
HDL-C	≥ 1 or 1.2 mmol/L^a^	1.6 (1.3, 1.9)	188	90	1.0 (0.9, 1.1)	24	45
Triglycerides	≤ 1.7 mmol/L	1.0 (0.7, 1.6)	188	83	2.1 (1.3, 2.9)	24	42
Systolic BP	140 mmHg	124 (119, 132)	197	95	126 (123, 130)	20	90
Diastolic BP	85 mmHg	70 (63, 75)	197	99	73 (67, 80)	20	90
BMI	≥ 18 to < 25 kg/m^2^	24 (22, 27)	187	54	30 (26, 33)	22	13

*TC* total cholesterol, *LDL-C* low-density lipoprotein cholesterol, *HDL-C* high-density lipoprotein cholesterol, *BP* blood pressure, *BMI b*ody mass index (kg/m^2^)

^a^≥1 mmol/L for males, ≥ 1.2 mmol/L for females

### Non-attendance at clinic appointments

Median non-attendance rate was 27 (0–50)%. More than one quarter of individuals (27%) missed more than half of all appointments. Higher non-attendance rate was positively correlated with a higher HbA1c (*r* = 0.3, *p* = < 0.001).

### Index of multiple deprivation (IMD)

We used the English Index of Multiple Deprivation 2015 (IMD) for each participant’s postcode from the Ministry of Housing, Communities and Local Government. (Available at: https://www.gov.uk/government/statistics/english-indices-of-deprivation-2015). Manchester Diabetes Centre serves an area of high social deprivation. Half of the cohort was in lowest quartile of the English Index of Multiple Deprivation (IMD ≤ 8211) and 79% were in the lower half of the distribution. Higher levels of deprivation were positively correlated with higher rates of non-attendance. (*r* = 0.15, *p* = 0.04). However, there was no correlation between IMD and HbA1c, BMI, total, LDL cholesterol, and systolic or diastolic blood pressure. There was an association of lower levels of HDL (*r* = 0.21, *p* = 0.002) in those with the higher levels of deprivation and a trend towards higher triglycerides (*r* = 0.14, *p* = 0.06).

## Discussion

In our study, only a small proportion of young adults achieved good glycaemic control. Non-attendance was high. Overweight and obesity were common with approximately half of the cohort having a BMI > 25 kg/m^2^. One-third had a TC > 5 mmol/L and one-fifth had LDL-cholesterol > 3 mmol/L. A combination of poor glycaemia, obesity, and dyslipidaemia can contribute to avoidable microvascular and macrovascular complications and poor long-term health outcomes.

The UK National paediatric diabetes audit 2015/16 reported similar HbA1c levels in 20–24 years old: mean HbA1c; men: 69 mmol/mol; women: 84 mmol/mol [3]. However, this report included only 26 individuals in that age range. Our study is considerably larger (*n* = 240) and, therefore, provides more precision in the estimate of glucose control. McKnight et al. published an international comparison of glycaemic control in T1DM in three age groups [4]. They reported a median HbA1c of 76 mmol/mol (9.1%) in 15–24 years old in England and Wales (*n* = 20,939). The USA Type 1 diabetes exchange investigators also evaluated HbA1c data in 2867 individuals aged 18–25 years [5]. Mean HbA1c was 8.7% compared to 9.0% this study.

Ours is the first study in the UK to report HbA1c, cardio-metabolic risk factors, and attendance in 18–25 years old specifically. Our findings may be generalizable to the other UK centers. Key limitation is the single-center retrospective nature of study. Other limitations include lack of information about rates of hypoglycaemia, hospital admissions due to diabetic ketoacidosis, and information about education provided to participants.

Our data highlight poor glycaemic control regardless of insulin delivery method, high rates of non-attendance, and other adverse cardio-metabolic risk factors among 18–25 years old with diabetes. There is an urgent need to further research the underlying reasons for these observations with a view to improve care and engagement with young adults who may benefit from new service delivery models.

## Abbreviations

BMI: Body mass index
BP: Blood pressure
HDL: High-density lipoprotein
IMD: Index of Multiple Deprivation 2015
LDL: Low-density lipoprotein
T1DM: Type 1 diabetes
T2DM: Type 2 diabetes
TC: Total cholesterol
